# 

**Published:** 1901-11

**Authors:** 


### Austin District Medical Society.

The Austin District Medical Society will hold its regular semi-annual meeting in Austin, December 19th and 20th (prox.). The annual banquet will be given on the evening of the 19th. An attractive program will be served up, hot. It is expected, requested and urged that every member will be present, and that each member will bring with him some friend or colleague, and have him join the society.

### The North Texas Medical Association.

The semi-annual meeting of the North Texas Medical Association will be held in Greenville, December 10th, 11th and 12. The officers are: Dr. R. F. Miller, Sherman, President; Dr. J. B. Shelmire, Dallas, Vice-President; Dr. S. B. Moore, Van Alstyne, Secretary; Dr. C. A. Gray, Bonham, Treasurer. The sections are in charge of the following: Surgery—Dr. Bacon Saunders, Fort Worth. Practice—Dr. R. W. Baird, Dallas. Obstetrics and Gynecology—Dr. J. B. Garrett, Gainesville. An attractive program will be presented. All reputable physicians are invited to attend. The North Texas Association is the largest Association in the State, except perhaps the State Medical Society. Its meetings are always well attended and the proceedings full of interest.

